# Eriodictyol alleviates ovarian dysfunction in a mouse model of premature ovarian failure via the PI3K/Akt/NF-κB pathway and suppression of macrophage inflammation

**DOI:** 10.3389/fphar.2025.1710805

**Published:** 2025-12-03

**Authors:** Miao Qu, Lusheng Liu, Jianwei Wang, Fangyu Sui, Miao Zhang, Xinyu Wu, Shixin Luo, Min Sun

**Affiliations:** 1 Department of Traditional Chinese Medicine, Basic Medicine College, Heilongjiang University of Traditional Chinese Medicine, Heilongjiang, China; 2 Department of Acupuncture and Moxibustion, Shanghai Traditional Chinese Medicine Integrated Hospital, Shanghai University of Traditional Chinese Medicine, Shanghai, China; 3 Graduate School, Heilongjiang University of Traditional Chinese Medicine, Heilongjiang, China

**Keywords:** premature ovarian failure, eriodictyol, PI3K/Akt/NF-κB, inflammation, macrophage, granulosa cell

## Abstract

**Background:**

Premature ovarian failure (POF) is a significant cause of female infertility characterized by amenorrhea, hypergonadotropism, and hypoestrogenism, for which effective treatments are limited. Eriodictyol, a natural flavonoid, possesses potent anti-inflammatory properties, but its effects on POF remain unexplored. This study aimed to investigate the therapeutic potential of eriodictyol in a mouse model of chemotherapy-induced POF and to elucidate its underlying molecular mechanism.

**Methods:**

A POF model was established in C57BL/6 mice by cyclophosphamide injection. Mice were then treated with eriodictyol (20, 40, or 80 mg/kg) for 4 weeks. Ovarian function was evaluated by estrous cyclicity, ovarian index, and serum hormone levels. The mechanism was investigated using a combination of computational prediction and experimental validation, including *in vivo* Western blotting and an *in vitro* macrophage-granulosa cell co-culture system.

**Results:**

Eriodictyol treatment markedly restored estrous cyclicity, increased the ovarian index, decreased serum follicle-stimulating hormone (FSH), and elevated serum estradiol (E2) and anti-Müllerian hormone (AMH) levels in POF mice. To explore the mechanism, network analysis was first employed to predict potential targets, which identified the PI3K/Akt/NF-κB signaling pathway. This computational hypothesis was then experimentally validated; Western blot analysis confirmed that eriodictyol significantly inhibited the phosphorylation of PI3K, Akt, and NF-κB p65 in ovarian tissues. Furthermore, molecular docking suggested a strong binding affinity between eriodictyol and Akt. Corroborating these findings, *in vitro* experiments demonstrated that eriodictyol pre-treatment of macrophages protected co-cultured granulosa cells from inflammatory damage.

**Conclusion:**

Eriodictyol alleviates chemotherapy-induced ovarian dysfunction by inhibiting the PI3K/Akt/NF-κB inflammatory pathway and suppressing macrophage-mediated damage to granulosa cells. These findings identify eriodictyol as a promising therapeutic candidate for POF.

## Introduction

Premature ovarian failure (POF), also known as premature ovarian insufficiency (POI), is a heterogeneous disorder defined by the loss of ovarian function before the age of 40 (Bai and Wang, 2022). Affecting approximately 1% of women worldwide, it presents with amenorrhea, hypergonadotropism, and hypoestrogenism, leading to infertility (Bricaire et al., 2013). The increasing prevalence of POF poses a significant public health challenge, as it is also associated with long-term health risks, including cardiovascular disease, osteoporosis, and cognitive decline, all of which diminish quality of life (Ghahremani-Nasab et al., 2020; Pal and Santoro, 2002; Vogt et al., 2022).

The etiology of POF is complex, encompassing genetic abnormalities, autoimmune disorders, and iatrogenic factors like chemotherapy (Pu et al., 2023). However, a majority of cases are idiopathic, with the precise cause remaining unknown (Tulandi and Kinch, 1981). The underlying pathogenesis is poorly understood but is thought to involve accelerated follicular depletion and atresia (Tuohy and Altuntas, 2007). Recently, chronic inflammation and oxidative stress have emerged as critical drivers of ovarian cellular damage and dysfunction in POF (Ding et al., 2024; Dragojević-Dikić et al., 2013; Fox, 1992; Yu et al., 2023).

Current management for POF primarily relies on hormone replacement therapy (HRT), which alleviates menopausal symptoms but fails to restore ovarian function or fertility (Armeni et al., 2021; Hamoda and Sharma, 2024). Moreover, long-term HRT is associated with increased risks of breast cancer and thromboembolic events, limiting its widespread use (Sullivan et al., 2016). Alternative strategies like stem cell therapy remain experimental, highlighting the urgent need for novel, safe, and effective therapeutic options for POF (Fàbregues et al., 2021).

In this context, botanical drugs and their bioactive metabolites have shown therapeutic promise due to their multi-target pharmacological profiles (Cai et al., 2021, Liu et al., 2022). For instance, Si-Wu-tang, a traditional botanical formulation, improved ovarian reserve in a mouse model of autoimmune POF (Liu et al., 2023), while Jinfeng Pill promoted follicle development in a rat model of chemically induced POF (Hu et al., 2023). These effects are often attributed to specific bioactive metabolites within the medicinal herbs, such as resveratrol, which has demonstrated protective effects against chemotherapy-induced ovarian damage (Hu et al., 2024; Jiang et al., 2019; Mobasher et al., 2022; Sevgin and Erguven, 2024). Modern computational tools, such as network analysis, can help to generate hypotheses regarding the complex mechanisms of these metabolites, often pointing towards pathways involved in hormone regulation, immune modulation, and anti-inflammation (Zhang et al., 2023; Zhao et al., 2023).

**FIGURE 1:**
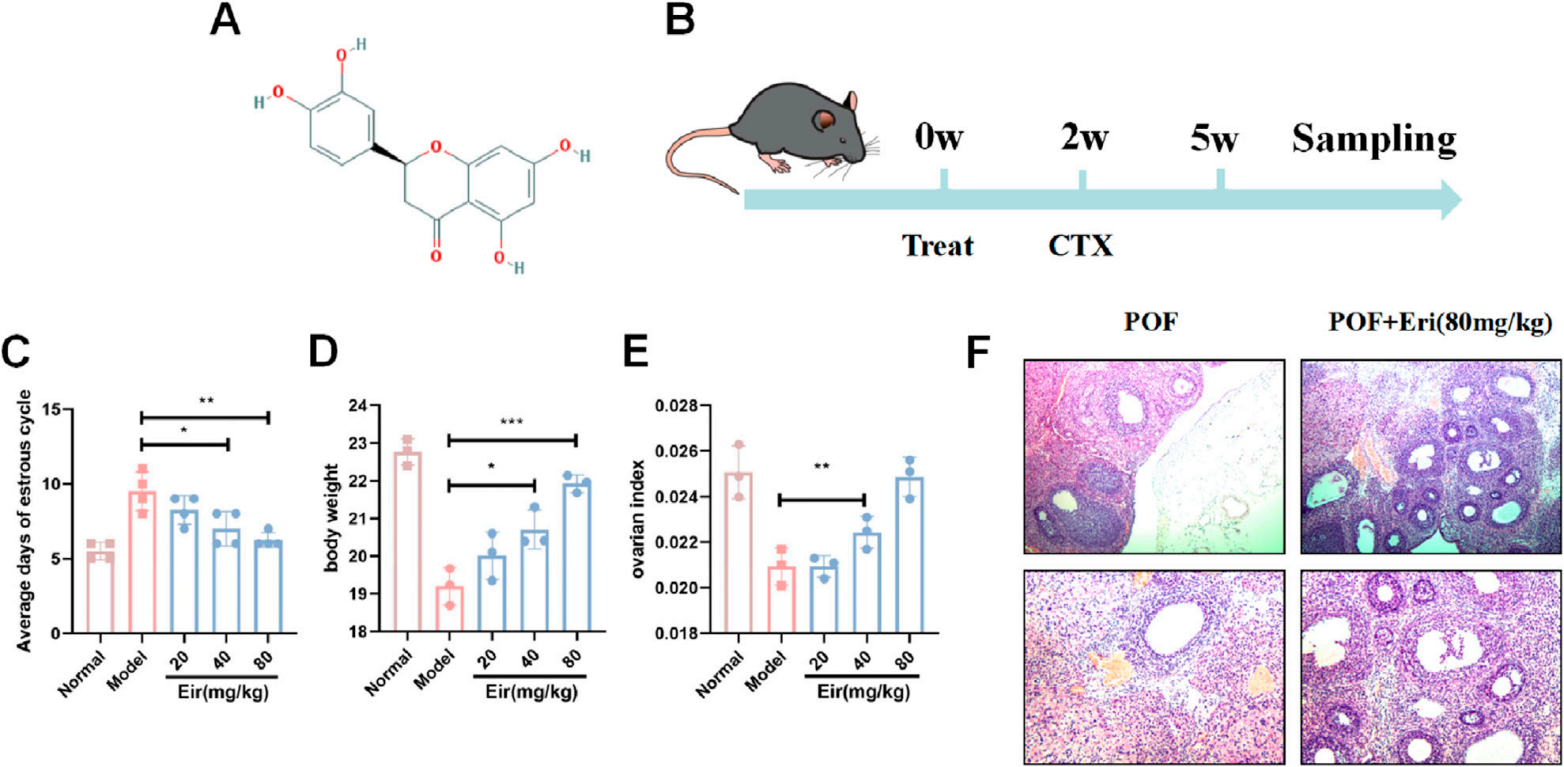
Eriodictyol improves estrous cycle and ovarian index in POF mice. **(A)** The molecular structure of eriodictyol. **(B)** The experimental design and workflow. **(C)** Eriodictyol treatment significantly shortened the average days of estrous cycle in POF mice. **(D)** Eriodictyol treatment significantly attenuated the decrease in body weight in POF mice. **(E)** Eriodictyol treatment significantly improved the decrease in ovarian index in POF mice. **(F)** HE staining showed that Eriodictyol treatment significantly improved the reduction in follicle count in POF mice Data are presented as mean ± SD. ##P < 0.01 vs. Control; *P < 0.05, **P < 0.01 vs. POF.

Eriodictyol (Figure 1A) is a natural flavanone found abundantly in citrus fruits and is a primary bioactive metabolite of the North American medicinal herb Yerba Santa (*Eriodictyon glutinosum* Benth.) (Vega-Villa et al., 2008; Zhu GF et al., 2015). Previous research has established its broad pharmacological activities, including potent anti-inflammatory, antioxidant, neuroprotective, and cardioprotective effects (Kang et al., 2024; Zheng et al., 2023). Its anti-inflammatory properties are particularly relevant, as eriodictyol has been shown to suppress inflammatory responses in macrophages by inhibiting key signaling pathways such as nuclear factor-κB (NF-κB) (He et al., 2019).

Despite the well-documented ovarian protective effects of other flavonoids like resveratrol (Hu et al., 2024; Jiang et al., 2019; Mobasher et al., 2022; Sevgin and Erguven, 2024), the potential role of eriodictyol in POF has not been investigated. This study is the first to systematically evaluate eriodictyol as a potential therapeutic agent for POF. Our work is distinguished by a focused mechanistic investigation designed to bridge eriodictyol’s known anti-inflammatory properties with ovarian pathophysiology. Specifically, we hypothesized that eriodictyol could alleviate POF by targeting inflammatory pathways.

Therefore, this study was designed to assess the therapeutic effects of eriodictyol in a cyclophosphamide (CTX)-induced mouse model of POF. We aimed to evaluate its impact on ovarian function, hormone profiles, and follicular dynamics. Furthermore, we sought to test the hypothesis, guided by predictions from our network analysis, that eriodictyol’s protective mechanism involves the inhibition of the PI3K/Akt/NF-κB signaling pathway and the suppression of macrophage-mediated inflammatory damage within the ovarian microenvironment.

## Materials and methods

### Animals and experimental design

Female C57BL/6 mice (8 weeks old, 20–25 g) were purchased from the Experimental Animal Center of Heilongjiang University of Traditional Chinese Medicine. Mice were housed in a specific pathogen-free (SPF) facility under a 12 h light/dark cycle with controlled temperature (22 °C ± 2 °C) and humidity (50% ± 10%). All animals had *ad libitum* access to standard chow and water. The study protocol was approved by the Institutional Animal Care and Use Committee of Heilongjiang University of Traditional Chinese Medicine (Approval No. 2025030517).

Following a 1-week acclimatization period, mice were randomly assigned to five groups (n = 10 per group): (1) Control, (2) POF model, (3) POF + Eriodictyol (20 mg/kg), (4) POF + Eriodictyol (40 mg/kg), and (5) POF + Eriodictyol (80 mg/kg). Eriodictyol (purity >98%; Nanjing Jiancheng Company, Nanjing, China) was suspended in 0.5% sodium carboxymethyl cellulose (CMC-Na) and administered daily by oral gavage for 28 consecutive days. The Control and POF model groups received an equivalent volume of the vehicle (0.5% CMC-Na). After 2 weeks of eriodictyol or vehicle administration, mice in the POF and eriodictyol treatment groups were intraperitoneally injected with cyclophosphamide (CTX; 120 mg/kg, once daily) for 14 consecutive days to induce POF. Control mice received an equivalent volume of physiological saline.

### Sample collection and hormone assays

At the conclusion of the experiment, mice were fasted overnight and then anesthetized with pentobarbital sodium (50 mg/kg, i.p.). Blood was collected via the retro-orbital plexus, and serum was isolated by centrifugation at 3,000 rpm for 15 min. Serum concentrations of follicle-stimulating hormone (FSH), luteinizing hormone (LH), estradiol (E2), and anti-Müllerian hormone (AMH) were quantified using commercial enzyme-linked immunosorbent assay (ELISA) kits (Nanjing Boyan Company, Nanjing, China) according to the manufacturer’s protocols.

Following blood collection, mice were euthanized by cervical dislocation. Ovaries were immediately excised, weighed, and processed for subsequent analysis. The left ovary from each mouse was fixed in 4% paraformaldehyde for histological examination, while the right ovary was snap-frozen in liquid nitrogen and stored at −80 °C for Western blot and quantitative real-time PCR (qRT-PCR) analyses.

### Vaginal cytology and estrous cycle evaluation

To assess the estrous cycle, vaginal smears were obtained daily (9:00–11:00 a.m.) from all mice during the last 2 weeks of the experiment. The vaginal cells were collected by gently flushing the vagina with 20 μL of normal saline, and the smears were air-dried, fixed with 95% ethanol, and stained with hematoxylin and eosin (H&E). The stage of the estrous cycle was determined based on the predominant cell types observed under a light microscope, including proestrus (nucleated epithelial cells), estrus (cornified squamous epithelial cells), metestrus (cornified squamous epithelial cells and leukocytes), and diestrus (predominantly leukocytes), according to established criteria (Byers et al., 2012).

### Ovarian histology and follicle counting

The fixed ovaries were dehydrated, embedded in paraffin, and serially sectioned at a thickness of 5 μm. The sections were stained with H&E for histological evaluation. The numbers of primordial, primary, secondary, and antral follicles were counted in every 5th section throughout the entire ovary by two independent observers blinded to the experimental groups. The follicles were classified according to the following criteria: primordial follicle (oocyte surrounded by a single layer of flattened granulosa cells), primary follicle (oocyte surrounded by a single layer of cuboidal granulosa cells), secondary follicle (oocyte surrounded by two or more layers of cuboidal granulosa cells without an antral cavity), and antral follicle (oocyte surrounded by multiple layers of cuboidal granulosa cells with an antral cavity), based on the classification system previously described (Pedersen and Peters, 1968).

### Western blot analysis

Based on the physiological and hormonal data, which demonstrated the most potent therapeutic effect at the 80 mg/kg dose, ovarian tissues from the Control, POF, and high-dose eriodictyol groups were selected for mechanistic analysis. Ovarian tissues were homogenized in RIPA lysis buffer (Beyotime, Shanghai, China) supplemented with protease and phosphatase inhibitor cocktails. Protein concentration was determined using a BCA Protein Assay Kit (Beyotime). Equal amounts of protein (30 μg per lane) were separated by 10% SDS-PAGE and transferred to polyvinylidene difluoride (PVDF) membranes (Millipore, MA, United States). Membranes were blocked with 5% non-fat milk in Tris-buffered saline with Tween 20 (TBST) for 1 h at room temperature. Subsequently, membranes were incubated overnight at 4 °C with the following primary antibodies: anti-phospho-PI3K, anti-PI3K, anti-phospho-Akt, anti-Akt, anti-phospho-NF-κB p65, anti-NF-κB p65, and anti-β-actin (all from Cell Signaling Technology, MA, United States). After washing with TBST, membranes were incubated with HRP-conjugated secondary antibodies (Beyotime) for 1 h at room temperature. Immunoreactive bands were visualized using an enhanced chemiluminescence (ECL) kit (Beyotime) and quantified using ImageJ software (NIH, MD, United States). β-actin served as the loading control.

### Quantitative real-time PCR

Total RNA was extracted from the ovarian tissues using TRIzol reagent (Invitrogen, CA, United States) according to the manufacturer’s protocol. The RNA concentration and purity were measured using a NanoDrop 2000 spectrophotometer (Thermo Fisher Scientific, MA, United States). Reverse transcription was performed using a PrimeScript RT reagent kit (Takara, Dalian, China). qRT-PCR was carried out on an ABI 7500 real-time PCR system (Applied Biosystems, CA, United States) using a TB Green Premix Ex Taq II kit (Takara). The primers used were as follows: AMH forward, 5′-GCA​GTT​GCT​AGT​CCT​ACA​TC-3′; AMH reverse, 5′-TCA​TCC​GCG​TGA​AAC​AGC​G-3′; β-actin forward, 5′-CAT​GTA​CGT​TGC​TAT​CCA​GGC-3′; β-actin reverse, 5′-CTC​CTT​AAT​GTC​ACG​CAC​GAT-3′. The relative mRNA expression levels were calculated using the 2^−ΔΔCT^ method and normalized to β-actin.

### In silico target prediction and network analysis

To generate hypotheses regarding the potential molecular mechanisms of eriodictyol, a network analysis was performed. Putative protein targets of eriodictyol were predicted using the SwissTargetPrediction (http://www.swisstargetprediction.ch/) and STITCH (http://stitch.embl.de/) databases. Genes associated with CTX-induced POF were retrieved from the GeneCards (https://www.genecards.org/) and DisGeNET (https://www.disgenet.org/) databases. A protein-protein interaction (PPI) network of the overlapping targets was constructed using the STRING database (https://string-db.org/) and visualized with Cytoscape software (version 3.8.0). Gene Ontology (GO) and Kyoto Encyclopedia of Genes and Genomes (KEGG) pathway enrichment analyses were performed using the clusterProfiler package in R (version 4.0.2) to identify potentially relevant biological pathways.

### Molecular docking

The 3D structure of eriodictyol was downloaded from the PubChem database (https://pubchem.ncbi.nlm.nih.gov/). The crystal structures of the key target proteins (PI3K, Akt, and NF-κB p65) were obtained from the RCSB Protein Data Bank (https://www.rcsb.org/). The proteins and ligand were prepared using AutoDock Tools (version 1.5.6). Molecular docking was performed using AutoDock Vina (version 1.1.2) to predict the binding affinity and interaction between eriodictyol and the target proteins. The docking results were visualized using PyMOL (version 2.4.0).

### Cell culture and treatment

Mouse macrophage cell line RAW264.7 and human granulosa cell line KGN were purchased from the Cell Bank of the Chinese Academy of Sciences (Shanghai, China). The cells were cultured in Dulbecco’s modified Eagle’s medium (DMEM, Gibco, CA, United States) supplemented with 10% fetal bovine serum (FBS, Gibco) and 1% penicillin-streptomycin (Gibco) at 37 °C in a humidified atmosphere with 5% CO2. For the co-culture experiment, RAW264.7 cells were seeded in the upper chamber of a transwell plate (0.4 μm pore size, Corning, NY, United States) and pretreated with eriodictyol (0, 12.5, or 25 μM) for 2 h, followed by stimulation with lipopolysaccharide (LPS, 1 μg/mL, Sigma-Aldrich, MO, United States) for 24 h. KGN cells were seeded in the lower chamber and co-cultured with the RAW264.7 cells for 24 h. The viability of KGN cells was assessed using a Cell Counting Kit-8 (CCK-8, Dojindo, Kumamoto, Japan) according to the manufacturer’s instructions.

### Statistical analysis

All data are presented as mean ± standard deviation (SD). Statistical analyses were performed using GraphPad Prism software (version 8.0). Comparisons among multiple groups were conducted by one-way analysis of variance (ANOVA) followed by Tukey’s *post hoc* test. A P-value <0.05 was considered statistically significant.

## Results

### Eriodictyol alleviates ovarian dysfunction and preserves ovarian reserve in POF mice

To evaluate the protective effect of eriodictyol on chemotherapy-induced ovarian damage, we first assessed key physiological and hormonal markers of ovarian function. Compared to control mice, the POF model group displayed significant ovarian dysfunction, characterized by prolonged and irregular estrous cycles (Figure 1C, P < 0.01). Treatment with eriodictyol, particularly at medium and high doses, markedly restored normal estrous cyclicity (P < 0.05). Furthermore, the POF model mice exhibited a significant reduction in body weight and ovarian index (Figures 1D,E, P < 0.01), which was dose-dependently attenuated by eriodictyol administration (P < 0.05). Histological analysis with H&E staining revealed a stark depletion of follicles in the POF group, whereas eriodictyol treatment effectively preserved the ovarian follicle pool (Figure 1F).

**FIGURE 2:**
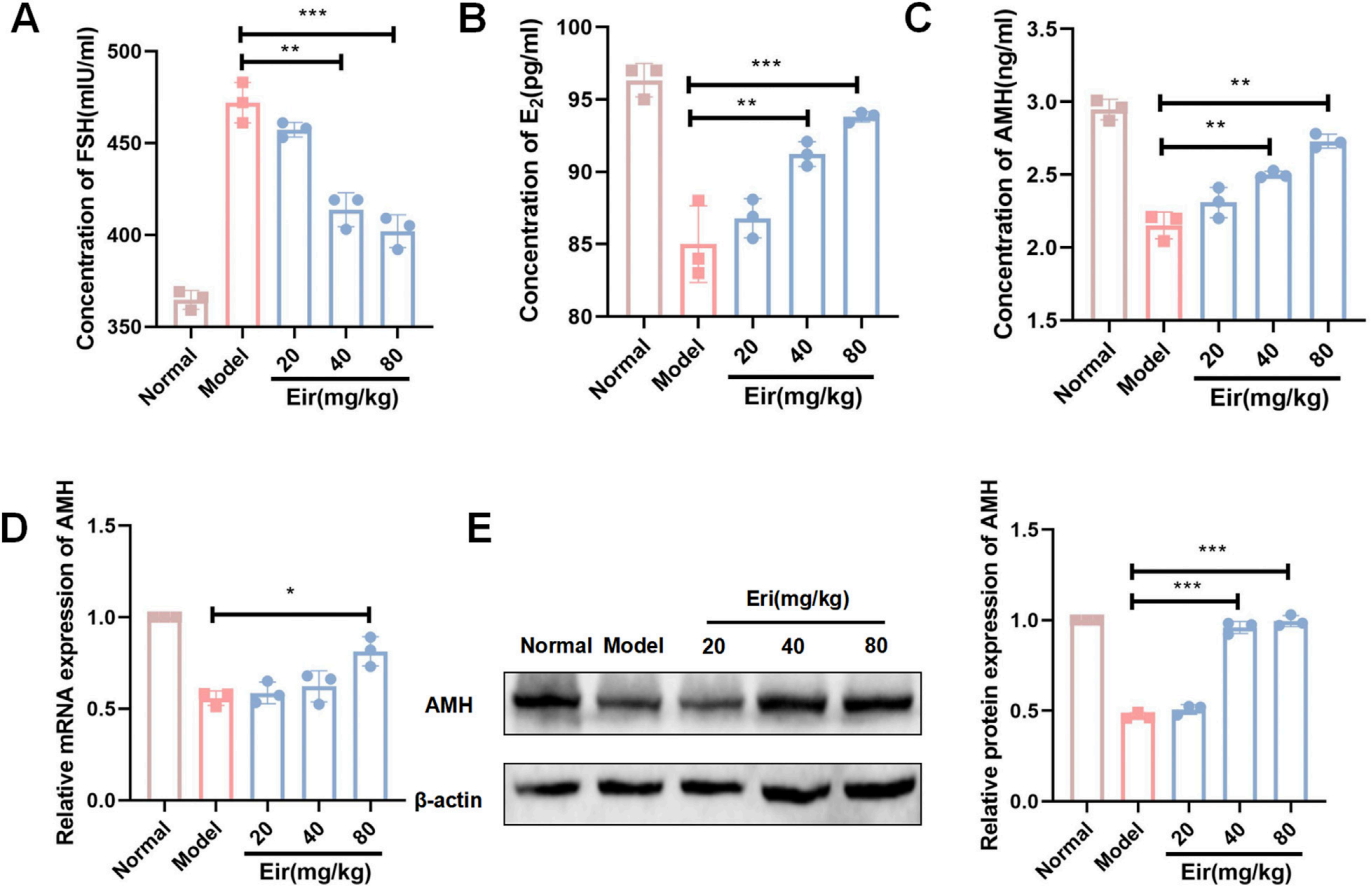
Eriodictyol improves serum hormone levels and ovarian AMH expression in POF mice. **(A)** Eriodictyol treatment significantly decreased the serum concentration of FSH in POF mice. **(B)** Eriodictyol treatment significantly increased the serum concentration of E2 in POF mice. **(C)** Eriodictyol treatment significantly increased the serum concentration of AMH in POF mice. **(D)** Eriodictyol treatment significantly upregulated the mRNA expression of AMH in ovarian tissues of POF mice. **(E)** Eriodictyol treatment significantly upregulated the protein expression of AMH in ovarian tissues of POF mice. Data are presented as mean ± SD. ##P < 0.01 vs. Control; *P < 0.05, **P < 0.01 vs. POF.

Consistent with these findings, serum hormone analysis showed that POF mice had elevated follicle-stimulating hormone (FSH) levels and decreased estradiol (E2) and anti-Müllerian hormone (AMH) levels (Figures 2A–C, P < 0.01). Eriodictyol treatment significantly reversed these hormonal imbalances, lowering FSH and increasing E2 and AMH concentrations in a dose-dependent manner (P < 0.05). To confirm the effect on ovarian reserve, we measured ovarian AMH expression. Both AMH mRNA and protein levels were significantly downregulated in the POF group but were substantially rescued by eriodictyol treatment (Figures 2D,E, P < 0.01).

### In silico network analysis predicts the PI3K/Akt pathway as a key therapeutic target

**FIGURE 3:**
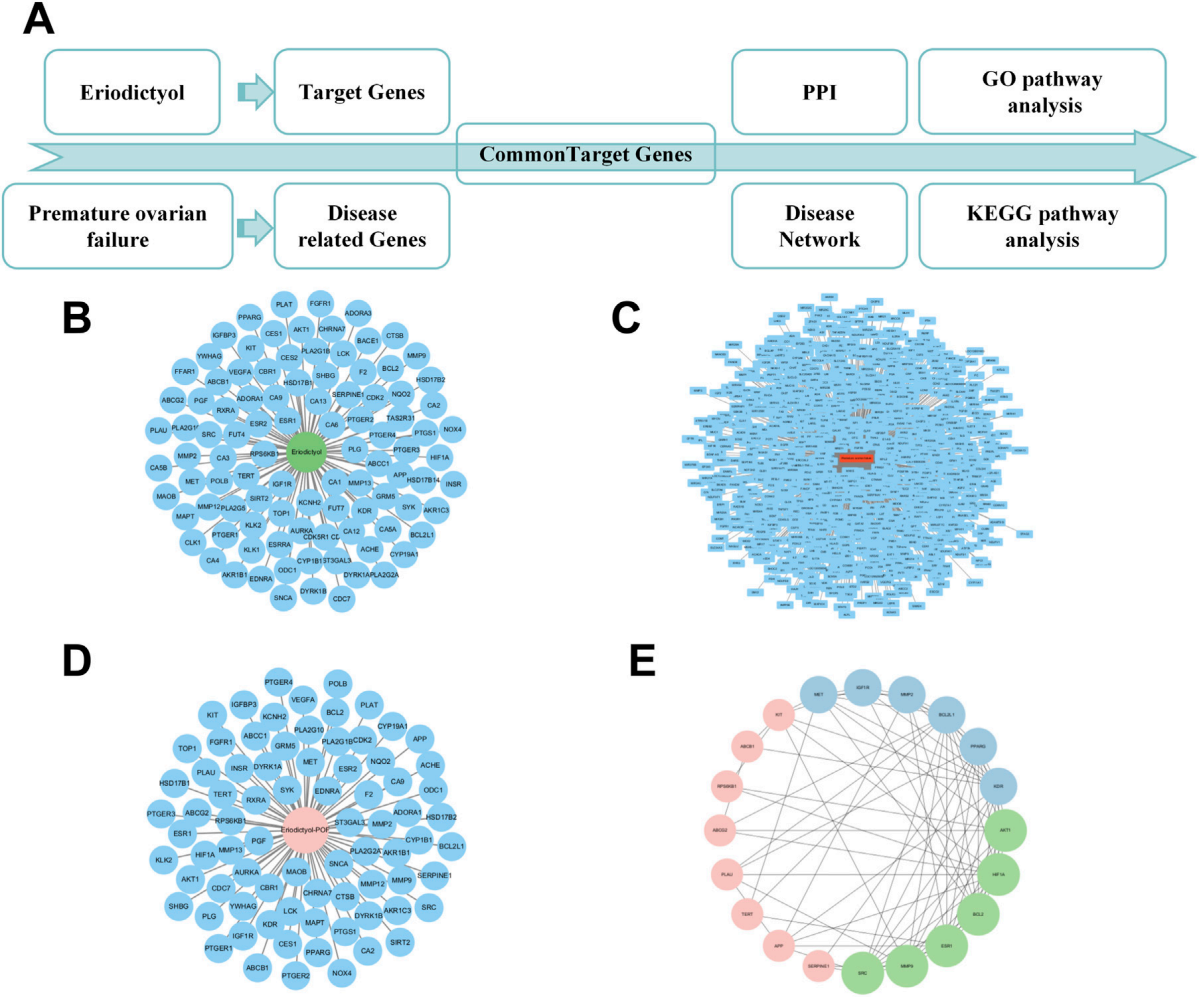
Network pharmacology analysis reveals the potential targets of eriodictyol in treating POF. **(A)** The workflow of network pharmacology analysis. **(B)** The putative targets of eriodictyol predicted by databases. **(C)** The POF-related genes collected from databases. **(D)** The overlapping genes between eriodictyol targets and POF-related genes. **(E)** The core targets in the PPI network of eriodictyol against POF.

**FIGURE 4:**
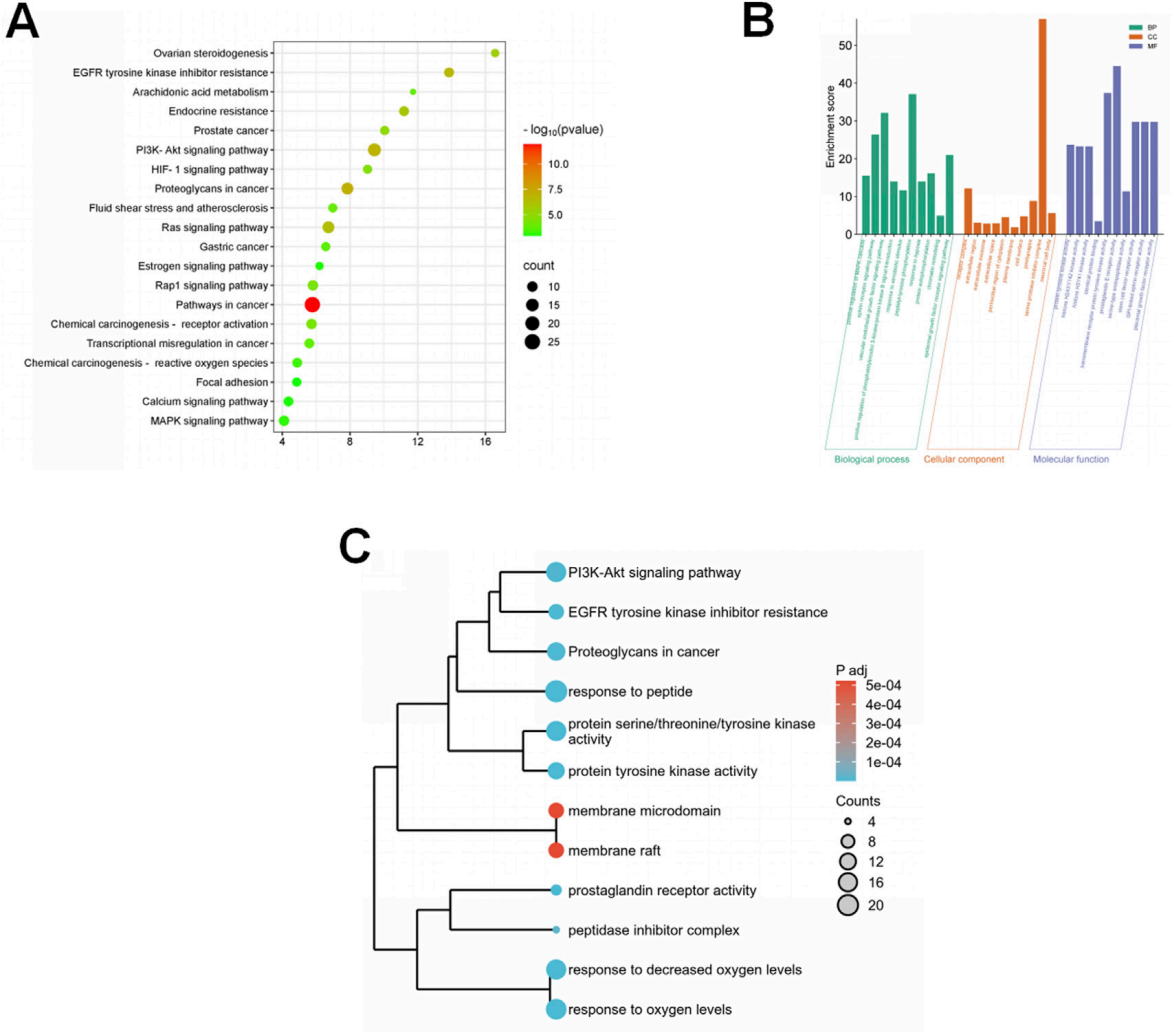
Bioinformatics analysis reveals the potential pathways of eriodictyol in treating POF. **(A)** KEGG pathway enrichment analysis showing the involvement of PI3K/Akt and MAPK inflammatory pathways. **(B)** GO enrichment analysis showing the involvement of inflammatory response and signal transduction. **(C)** The integrated analysis of KEGG and GO using a dendrogram plot.

To generate hypotheses about the molecular mechanism underlying eriodictyol’s protective effects, we performed an *in silico* network analysis (Figure 3A). We first identified putative protein targets for eriodictyol and genes associated with POF from public databases (Figures 3B,C). By intersecting these datasets, we obtained a list of potential therapeutic targets (Figure 3D). A protein-protein interaction (PPI) network was constructed, which highlighted several core hub genes, including AKT1, MAPK1, and MAPK3 (Figure 3E). Subsequent KEGG pathway enrichment analysis of these targets strongly predicted that the PI3K/Akt and MAPK signaling pathways were central to eriodictyol’s mechanism of action (Figure 4A). GO enrichment analysis further implicated biological processes related to inflammatory response and signal transduction (Figures 4B,C).

### Eriodictyol inhibits the activation of the PI3K/Akt/NF-κB pathway in ovarian tissue

**FIGURE 5:**
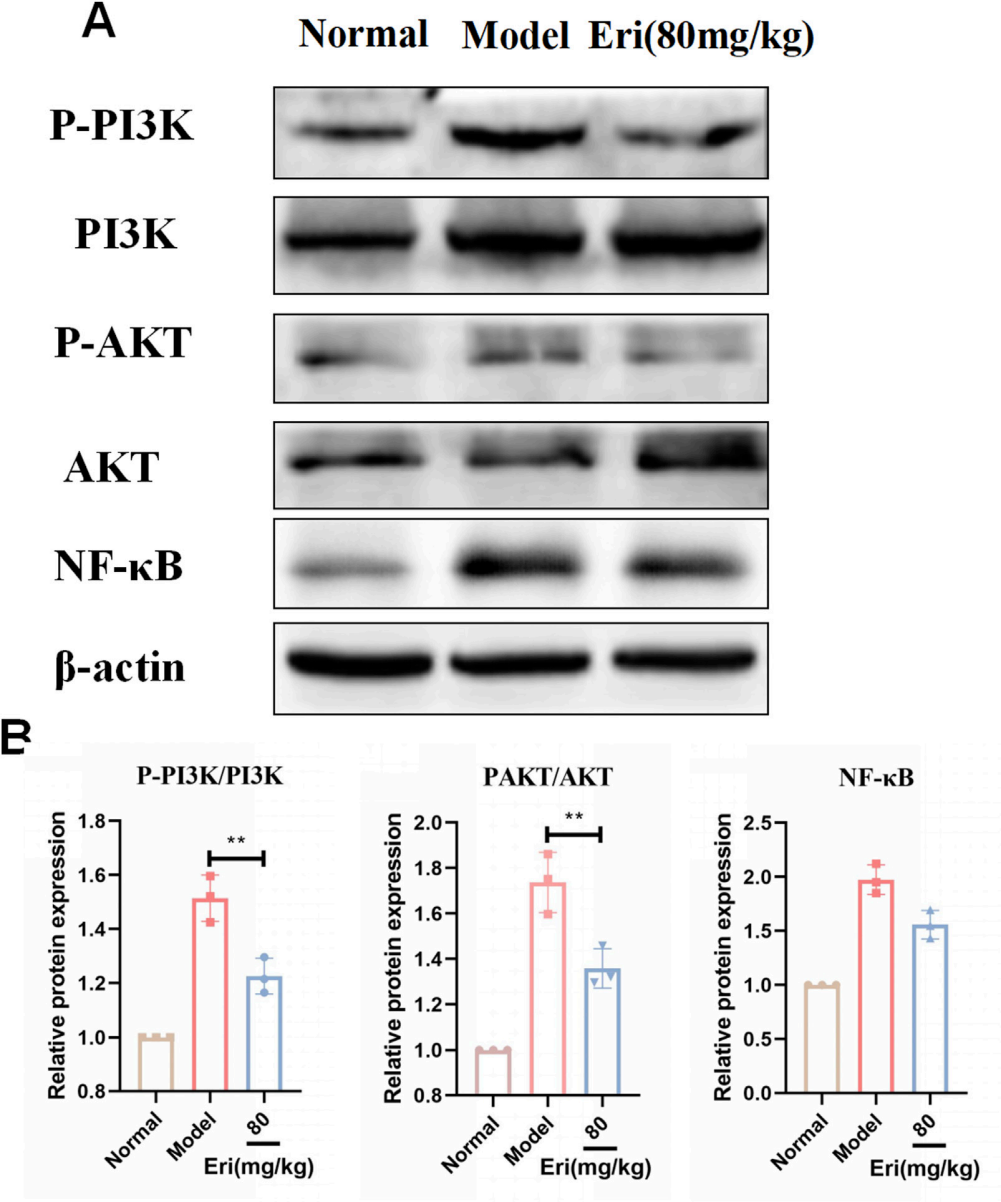
Eriodictyol modulates the PI3K/Akt/NF-κB signaling pathway in ovaries of POF mice. **(A)** Representative Western blot images showing the protein levels of p-PI3K, PI3K, p-Akt, Akt, p-NF-κB p65, and NF-κB p65 in ovarian tissues. **(B)** Quantitative analysis of the phosphorylation levels of PI3K, Akt, and NF-κB p65. Data are presented as mean ± SD (n = 3). *P < 0.05, **P < 0.01 vs. POF.

To experimentally validate the computational predictions, we examined the activation status of the PI3K/Akt/NF-κB pathway in ovarian tissues using Western blot. As shown in Figure 5A, the phosphorylation levels of PI3K, Akt, and the downstream inflammatory mediator NF-κB p65 were significantly elevated in the POF model group compared to controls (P < 0.01), indicating pathway activation during ovarian injury. In contrast, treatment with eriodictyol (80 mg/kg) markedly suppressed the phosphorylation of PI3K, Akt, and NF-κB p65 (P < 0.05). These results confirm that eriodictyol exerts its therapeutic effect by inhibiting this pro-inflammatory signaling cascade.

### Eriodictyol shows binding potential to Akt and protects granulosa cells from macrophage-induced inflammation

**FIGURE 6:**
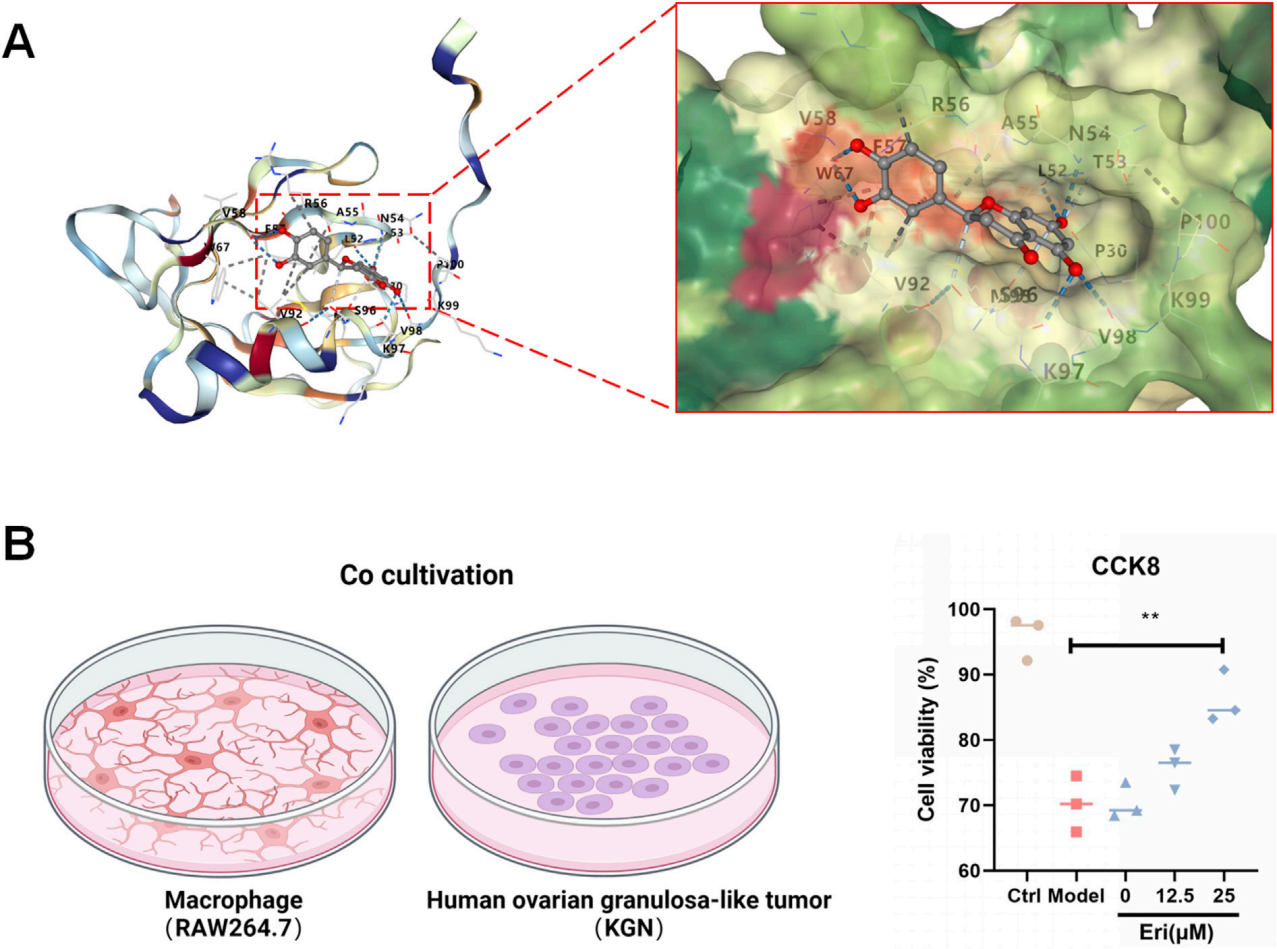
Eriodictyol directly binds to Akt and protects granulosa cells from inflammatory damage. **(A)** Molecular docking analysis showing that eriodictyol could directly bind to the kinase domain of Akt. **(B)** In a co-culture system, pretreatment of RAW264.7 macrophages with different concentrations of eriodictyol significantly attenuated the decrease in cell viability of KGN human granulosa cells induced by macrophage-mediated inflammatory damage. Data are presented as mean ± SD (n = 3). ##P < 0.01 vs. Control; *P < 0.05, **P < 0.01 vs. LPS.

To further explore the interaction with the validated pathway, molecular docking analysis was performed. The results suggested a strong binding affinity between eriodictyol and the kinase domain of Akt, indicating a potential direct inhibitory interaction (Figure 6A). To translate this finding to a cellular context, we established an *in vitro* co-culture model to simulate the inflammatory ovarian microenvironment. As shown in Figure 6B, when KGN granulosa cells were co-cultured with LPS-activated RAW264.7 macrophages, their viability was significantly reduced (P < 0.01). However, pre-treating the macrophages with eriodictyol (12.5 and 25 μM) dose-dependently protected the KGN cells from this inflammatory damage, significantly restoring their viability (P < 0.05). This demonstrates that eriodictyol can suppress macrophage-mediated inflammation and protect granulosa cells.

## Discussion

In the present study, we provide the first evidence that eriodictyol, a natural flavonoid, can ameliorate chemotherapy-induced POF in a mouse model. Our results demonstrate that eriodictyol administration effectively restored ovarian function, normalized serum hormone levels, and preserved the follicular pool. Mechanistically, we show that these protective effects are mediated through the inhibition of the PI3K/Akt/NF-κB signaling pathway and the suppression of macrophage-driven inflammation, which in turn protects granulosa cells from damage. These findings identify eriodictyol as a promising therapeutic candidate for POF.

From a pharmaceutical standpoint, understanding the pharmacokinetic profile of eriodictyol is crucial for its potential clinical translation (Badi et al., 2024). Following oral administration, eriodictyol is generally reported to be rapidly absorbed, with peak plasma concentrations typically reached within 1–2 h. However, like many flavonoids, it is subject to low oral bioavailability due to extensive first-pass metabolism in the intestine and liver (Li et al., 2016). The primary metabolic pathways include glucuronidation and sulfation, which convert eriodictyol into more water-soluble conjugates that are then circulated and excreted. It is important to consider that some of these metabolites may retain biological activity, potentially contributing to the overall therapeutic effects observed. The effective doses used in our study are consistent with those in other preclinical models and likely achieve systemic concentrations of eriodictyol and its metabolites sufficient to exert a pharmacological effect on ovarian tissue. Nonetheless, its limited bioavailability represents a key challenge that could be addressed through advanced formulation strategies, such as nano-encapsulation or the use of absorption enhancers, to improve its therapeutic efficacy in future clinical applications.

Chemotherapy-induced ovarian damage is a primary cause of POF in young female cancer survivors. Cyclophosphamide (CTX), a widely used alkylating agent, inflicts ovarian toxicity by directly damaging oocytes and granulosa cells, and by inducing oxidative stress and inflammation (Lee et al., 2022). Our CTX-induced POF model successfully recapitulated the key clinical features of the condition, including a disrupted estrous cycle, reduced ovarian index, and characteristic hormonal imbalances. Treatment with eriodictyol dose-dependently reversed these pathological changes, confirming its potent protective effect on ovarian function and reserve (Delkhosh et al., 2019).

AMH is a sensitive biomarker for ovarian reserve, and its levels are inversely correlated with the severity of POF (Alipour et al., 2015). In our study, the significant decrease in both serum and ovarian AMH in POF mice was robustly rescued by eriodictyol. This result, consistent with the observed preservation of follicles, suggests that eriodictyol’s therapeutic action involves maintaining the primordial follicle pool and supporting follicle development (Jiao et al., 2021).

To elucidate the underlying molecular mechanism, we adopted a strategy combining computational prediction with experimental validation. Our initial network analysis served as a hypothesis-generating tool, predicting that the PI3K/Akt and MAPK signaling pathways were key targets for eriodictyol. The PI3K/Akt pathway is a master regulator of cell survival and proliferation, and its aberrant activation is linked to ovarian disorders, including POF (Jiao et al., 2021; Han et al., 2022). NF-κB, a key downstream effector of this pathway, orchestrates inflammatory responses and is known to be activated in the ovaries of POF models. Critically, our Western blot analysis experimentally confirmed this hypothesis: we observed a marked increase in the phosphorylation of PI3K, Akt, and NF-κB p65 in the ovaries of POF mice, which was significantly suppressed by eriodictyol treatment. This demonstrates that eriodictyol’s anti-inflammatory effect in the ovary is mediated, at least in part, by downregulating the PI3K/Akt/NF-κB signaling cascade.

It is crucial, however, to acknowledge the inherent limitations of *in silico* methodologies. Network analysis and molecular docking are valuable for identifying potential targets and generating hypotheses but do not constitute definitive proof of a mechanism, as the underlying databases can contain false-positive interactions. Furthermore, as a flavonoid, eriodictyol could be classified as a pan-assay interference substance (PAIN), a class of molecules known to produce non-specific results in some high-throughput *in vitro* screens. Our study was designed to mitigate these concerns. We used the computational predictions strictly as a guide, with the core of our mechanistic claim resting on robust *in vivo* and *in vitro* experimental validation. The significant therapeutic effects observed in the whole-animal model, coupled with the specific protective effects on granulosa cells in a targeted co-culture system, provide compelling evidence for a specific biological activity rather than a non-specific artifact. The experimental confirmation of the PI3K/Akt/NF-κB pathway’s inhibition *in vivo* thus lends strong support to our initial computational prediction.

Our molecular docking analysis suggested a potential direct binding interaction between eriodictyol and the kinase domain of Akt. This is plausible and consistent with reports on other flavonoids, such as quercetin and baicalein (Han et al., 2022; Zheng et al., 2022), and may provide a molecular basis for its inhibitory action on the PI3K/Akt/NF-κB pathway.

Granulosa cells are essential for follicular development, and their apoptosis is a key event in atresia and POF. Ovarian macrophages contribute significantly to the local inflammatory microenvironment that can drive this cell death (Zheng et al., 2022; Ai et al., 2023). Our *in vitro* co-culture system, which mimics this inflammatory crosstalk, demonstrated that eriodictyol protects granulosa cells by acting on macrophages to quell the inflammatory response. This finding directly links the pathway inhibition observed *in vivo* to a tangible, protective cellular outcome and reinforces the idea that eriodictyol’s primary mechanism is the suppression of inflammation T.

While our findings position eriodictyol as a promising therapeutic agent, a thorough consideration of its safety profile is essential, especially when compared to current standard treatments like hormone replacement therapy (HRT). HRT, while effective for symptom management, is associated with significant long-term risks, including an increased incidence of breast cancer, thromboembolism, and stroke, which limits its long-term use and acceptance by many patients.

In contrast, eriodictyol, like many flavonoids, is generally considered to have a favorable safety profile. It is a natural metabolite found in various fruits and medicinal herbs and has been part of the human diet for centuries without significant reported toxicity. This is largely supported by preclinical toxicology studies, which have shown a lack of adverse effects even at doses substantially higher than those used in our study (Bai et al., 2019). Nevertheless, potential considerations should be acknowledged. At very high concentrations, some flavonoids may induce mild gastrointestinal discomfort. Furthermore, like other polyphenolic metabolites, eriodictyol could potentially interact with drug-metabolizing enzymes, such as cytochrome P450, which might affect the metabolism of concomitant medications (Robertson et al., 2015). While eriodictyol also possesses weak phytoestrogenic activity, this effect is typically orders of magnitude lower than that of the potent estrogens used in HRT and may even confer protective, rather than harmful, effects on hormone-sensitive tissues.

Crucially, this potential side-effect profile differs fundamentally from the severe, well-documented risks associated with HRT, such as thromboembolism and certain cancers. The concerns regarding eriodictyol at therapeutic doses are largely theoretical and center on potential drug interactions, whereas the risks of HRT are established and can be life-threatening. Therefore, eriodictyol represents a potentially safer alternative for managing POF, particularly for long-term use or in women with contraindications to HRT. However, rigorous clinical trials are imperative to definitively establish the long-term safety and efficacy of eriodictyol in humans.

Despite these promising findings, it is important to acknowledge several limitations of the present study. First, our investigation was conducted over a 4-week treatment period, which, while sufficient to demonstrate short-term protective effects, does not inform on the long-term efficacy or safety of eriodictyol. It remains to be determined whether the observed dose-dependent relationship would be sustained over extended periods. Future long-term studies are thus required to validate the therapeutic durability of eriodictyol.

Second, our experimental design assessed the protective effect of eriodictyol, as the flavonoid was administered prior to and during the induction of ovarian damage by cyclophosphamide. This model is highly relevant for fertility preservation in patients scheduled to undergo chemotherapy. However, it does not address whether eriodictyol can act therapeutically to restore function in an already-damaged ovary. A critical next step will be to investigate the effects of eriodictyol administration after POF has been established, which would assess its potential for treating existing idiopathic POF—a major clinical challenge. While these questions were beyond the scope of our initial investigation, they represent crucial avenues for future research.

## Conclusion

This study provides the first evidence for the therapeutic effects of eriodictyol on chemotherapy-induced POF. Our findings demonstrate that eriodictyol significantly alleviates ovarian dysfunction in a mouse model by restoring estrous cyclicity, normalizing hormone levels, and preserving the ovarian follicle pool. Mechanistically, we have shown that these protective effects are mediated through the inhibition of the PI3K/Akt/NF-κB inflammatory signaling pathway. Furthermore, eriodictyol directly protects granulosa cells from macrophage-mediated inflammatory damage. Collectively, these results highlight eriodictyol as a promising and potentially safer natural therapeutic candidate for the prevention and treatment of POF, warranting further clinical investigation.

## Data Availability

The data that support the findings of this study are available from the corresponding author upon reasonable request.
